# Study on the Impact of Dietary Patterns on Cardiovascular Metabolic Comorbidities among Adults

**DOI:** 10.21203/rs.3.rs-4451883/v1

**Published:** 2024-06-03

**Authors:** Danhui Mao, Gongkui Li, Yajing Li, Shixun Wang, Mohan Zhang, Mingyan Ma, Xiaojun Ren

**Affiliations:** Third Hospital of Shanxi Medical University, Shanxi Academy of Medical Sciences, Tongji Shanxi Hospital; Third Hospital of Shanxi Medical University, Shanxi Academy of Medical Sciences, Tongji Shanxi Hospital; Third Hospital of Shanxi Medical University, Shanxi Academy of Medical Sciences, Tongji Shanxi Hospital; Shanxi Medical University; Shanxi Medical University; Shanxi Medical University; Third Hospital of Shanxi Medical University, Shanxi Academy of Medical Sciences, Tongji Shanxi Hospital

**Keywords:** dietary pattern, cardiometabolic multimorbidity, latent profile analysis, adults

## Abstract

**Background:**

The prevalence of cardiovascular metabolic comorbidities (CMM) among adults is relatively high, imposing a heavy burden on individuals, families, and society. Dietary patterns play a significant role in the occurrence and development of CMM. This study aimed to identify the combined types of CMM in adult populations and investigate the impact of dietary patterns on CMM.

**Methods:**

Participants in this study were from the sixth wave of the China Health and Nutrition Survey (CHNS). Dietary intake was assessed using a three-day 24-hour dietary recall method among 4,963 participants. Latent profile analysis was used to determine dietary pattern types. Two-step cluster analysis was performed to identify the combined types of CMM based on the participants’ conditions of hyperuricemia, dyslipidemia, diabetes, renal dysfunction, hypertension, and stroke. Logistic regression analysis with robust standard errors was used to determine the impact of dietary patterns on CMM.

**Results:**

Participants were clustered into three dietary patterns (Pattern 1 to 3) and five CMM types (Class I to V). Class I combined six diseases, with a low proportion of diabetes. Class II also combined six diseases but with a high proportion of diabetes. Class III combined four diseases, with a high proportion of hypertension. Class IV combined three diseases, with the highest proportions of hyperuricemia, diabetes, and renal dysfunction. Class V combined two diseases, with high proportions of dyslipidemia and renal dysfunction. Patients with Class III CMM had a significantly higher average age than the other four classes (*P* ≤ 0.05). Compared to those with isolated dyslipidemia, individuals with a low-grain, high-fruit, milk, and egg (LCHFM) dietary pattern had a higher risk of developing dyslipidemia combined with renal dysfunction (Class V CMM) with an odds ratio of 2.001 (95% *CI* 1.011–3.960, *P*≤ 0.05).

**Conclusion:**

For individuals with isolated dyslipidemia, avoiding a low-grain, high-fruit, milk, and egg (LCHFM) dietary pattern may help reduce the risk of developing dyslipidemia combined with renal dysfunction (Class V CMM).

## Background

Cardiovascular metabolic comorbidities (CMM) refers to the coexistence of two or more cardiovascular diseases or metabolic disorders in an individual, such as hypertension, diabetes, dyslipidemia, and stroke [1]. It is one of the most stable patterns of multimorbidity [2]. In a study by Chudasama et al. (2019), approximately 64%, 59%, 57%, and 54% of patients with diabetes, angina pectoris, stroke, and myocardial infarction were concurrently diagnosed with hypertension [3]. A 10-year follow-up study conducted by Zhao et al. in 2021 on a Chinese population of 461,047 individuals revealed that 18.7% of healthy individuals experienced their first onset of heart disease, stroke, and diabetes. Among these newly diagnosed patients, 16.2% further progressed to CMM, and 22.5% of these patients died [4]. An epidemiological survey in 2022 showed that the incidence of CMM was 14.4%. CMM can significantly impact disease prognosis and patients’ quality of life, with studies indicating that the mortality risk among CMM patients is twice that of patients without multimorbidity [5–7]. As the population ages, the long duration and complex etiology of CMM pose greater risks of disability, death, as well as physical, psychological, and economic burdens on patients [7, 8]. Therefore, preventing and controlling CMM is a critical task in primary healthcare services.

According to statistics, CMM is closely associated with demographic characteristics. Research indicates that female gender, middle and old age, urban residency, and higher education levels are demographic risk factors for CMM [9]. Additionally, similar to single chronic diseases, studies have shown that smoking, excessive alcohol consumption, unhealthy dietary patterns, and physical inactivity are lifestyle risk factors for CMM [10, 11]. Diet, as one of the modifiable lifestyle factors, can be specifically adjusted to reduce disease risk. In terms of cardiovascular diseases, reducing saturated fat intake and increasing dietary fiber intake can lower the risk of developing cardiovascular diseases. High-energy-density diets and obesity increase the risk of cardiovascular diseases. Appropriate intake of dietary fiber aids in insulin secretion, glucose control, blood lipid levels, and blood pressure maintenance at healthy levels. Furthermore, dietary patterns with high-energy density, high saturated fat, and low dietary fiber are associated with increased cardiometabolic risks in severely obese individuals [11]. Regarding metabolic diseases, reducing macronutrient intake can reduce the risk of developing metabolic diseases. High-energy-density diets and obesity also increase the risk of metabolic diseases. Appropriate intake of dietary fiber, vitamins, and minerals benefits insulin secretion, glucose control, and blood lipid levels, maintaining them at healthy levels and reducing the risk of metabolic diseases [12–14]. Moreover, previous studies have found that certain dietary patterns can simultaneously influence the cardiovascular or metabolic systems, such as the Mediterranean diet, the Dietary Approaches to Stop Hypertension (DASH) diet, and the Atkins diet, which can affect body weight and serum uric acid levels [15–17].

CMM exhibits a complex disease mechanism, with diseases being highly interrelated. To prevent and control CMM, it is essential to fully consider the correlations between disease combinations and explore preventive and therapeutic measures from a holistic perspective. As a qualitative approach for CMM, the combination types of CMM can represent the complex relationships between diseases in a holistic manner. Meanwhile, as modifiable factors in preventing and controlling multiple cardiovascular diseases or metabolic disorders, foods or nutrients coordinate their actions in a dynamic and complex system. Therefore, evaluating diet also requires a comprehensive consideration of the intricate relationships between foods and nutrients, analyzed from a holistic angle. Dietary patterns, as a qualitative approach for dietary habits, can represent the complex relationships between foods or nutrients in a holistic manner. Thus, understanding the impact of dietary patterns on CMM combination types may be of significant importance for the prevention and control of CMM. Currently, studies on the effects of dietary patterns on cardiovascular or metabolic diseases primarily focus on the relationships between single diseases and single dietary patterns, or multiple diseases and a single dietary pattern. However, there is a lack of exploration into the relationships between various CMM combination types and multiple dietary patterns within a population. Therefore, this study aims to determine the impact of major dietary patterns on primary CMM combination types, based on an understanding of the primary CMM combination types and major dietary patterns within the population.

## Materials and Methods

### Study population

The participants in this study were derived from the sixth round of the China Health and Nutrition Survey (CHNS). For a detailed description of CHNS, please refer to https://www.cpc.unc.edu/projects/china. We excluded individuals with incomplete data, including socio-demographic characteristics, dietary and lifestyle habits, and disease characteristics. Therefore, the study encompassed a total of 4963 subjects (Table 1). The survey was approved by the ethics committee of the University of North Carolina at Chapel Hill and the National Institute for Nutrition and Health, Chinese Center for Disease Control and Prevention.

### Dietary Assessment

For this study, ten types of foods were selected based on their daily average intake exceeding 10g and their common occurrence in research on adult dietary patterns, see Table 4 for details.

### Disease Assessment

In this study, we assessed the presence of hyperuricemia, dyslipidemia, diabetes, chronic kidney disease, and hypertension among participants. Hyperuricemia was defined as serum uric acid levels above 420 μmol/L (7 mg/dl) for males and above 357 μmol/L (6 mg/dl) for females [18]. Dyslipidemia was defined as having at least one of the following conditions: (1) hypercholesterolemia (TC ≥ 5.18 mmol/L); (2) hypertriglyceridemia (TG ≥ 1.70 mmol/L); (3) low-density lipoprotein cholesterol (LDL-C) ≥ 3.37 mmol/L; and (4) high-density lipoprotein cholesterol (HDL-C) < 1.04 mmol/L [19]. Chronic kidney disease was identified as having at least one of the following conditions: (1) kidney dysfunction (abnormal kidney structure or function) lasting for more than 3 months; (2) glomerular filtration rate < 60 mL/min/1.73 m2 lasting for more than 3 months [20]. Diabetes was diagnosed if participants had either (1) fasting plasma glucose ≥ 7.0 mmol/L or 2-hour postprandial glucose ≥ 11.1 mmol/L; or (2) a prior diagnosis of diabetes by a physician [21]. Hypertension was defined as having either (1) an average systolic blood pressure > 140 mmHg or an average diastolic blood pressure > 90 mmHg; or (2) a prior diagnosis of hypertension by a physician [22].

### Measurements of covariates

The control variables in this study included demographic characteristics such as age and gender, as well as lifestyle features such as smoking status and physical activity. Smoking status was categorized as current or past smoker (yes = 1) and never smoker (no = 0). Additionally, we calculated the total weekly metabolic equivalents (MET) for physical activities, encompassing household, occupational, transportation, and recreational activities [23–25].

### Statistical analysis

Continuous variables, such as age, urbanization index, and physical activity, were described using mean ± standard deviation. Categorical variables, including gender, marital status, educational level, smoking status, and the presence of hypertension, stroke, diabetes, dyslipidemia, and renal dysfunction, were presented as frequencies (proportions). The differences in the distribution of demographic characteristics and disease status were analyzed using the *t*test, the *F*test, and the χ^2^ test, with subsequent Bonferroni corrections for multiple comparisons. Latent Profile Analysis (LPA) was employed to identify subgroups of individuals based on their dietary patterns, and Two-Step Clustering (TSC) was used to categorize individuals according to their disease conditions. Finally, robust standard error Logistic regression models were applied to determine the influence of dietary patterns on chronic metabolic diseases (CMM). All tests were conducted using a two-sided significance level of 95%.

## Results

### Demographic characteristics

A total of 4963 participants were included in this study, with 2658 females (53.6%) and 2305 males (46.4%). The participants’ ages ranged from 18 to 94 years, with a mean age of 51 ± 15 years. Among all participants, 69.6% were from rural areas, 77.1% had an education level below junior high school, and 5.7% were never married. The average urbanization index of the participants’ communities was 67.57 ± 19.01. The detailed information is presented in Table 5.

### Disease Status and CMM Combination Types

Two-Step Clustering (TSC) analysis was applied to categorize the disease status of the participants. Table 1 lists the fitting parameters of 15 different models (Models 1 to 15). Both Model 2 and Model 7 exhibited relatively high target distance measurement ratios (TDMR). However, Model 7 had a lower Bayesian Information Criterion (BIC) value compared to Model 2, indicating that Model 7 provided a superior classification ratio compared to the other models.

### Disease Prevalence Among Participants

The prevalence of hyperuricemia, dyslipidemia, diabetes, renal dysfunction, hypertension, and stroke among the participants is presented in Table 2. Specifically, 16.7% belonged to Class I, 9.9% to Class II, 15.0% to Class III, 8.8% to Class IV, 12.7% to Class V, 19.7% to Class VI, and 17.5% to Class VII.

Participants with Class I and II disease patterns were characterized by having all six diseases to varying degrees. The multiple comparisons revealed that those with Class I were most likely to have stroke, followed by a relatively high probability of hyperuricemia, dyslipidemia, and renal dysfunction, but a lower likelihood of diabetes. In contrast, those with Class II showed a higher probability of dyslipidemia, diabetes, and renal dysfunction, but a lower likelihood of stroke. They also had a relatively high probability of hyperuricemia, dyslipidemia, and renal dysfunction, but a lower probability of hyperuricemia, hypertension, and stroke. Participants in Class III presented a pattern of having four diseases, with the highest likelihood of hypertension and moderate probabilities of dyslipidemia, renal dysfunction, and stroke. Class IV participants had three diseases, with the highest probabilities of hyperuricemia, diabetes, and renal dysfunction. Participants in Class V exhibited a two-disease pattern, with the highest likelihood of dyslipidemia and a higher probability of renal dysfunction. Both Classes VI and VII were characterized by having only one disease, with Class VI showing a relatively higher probability of dyslipidemia and Class VII a relatively higher probability of stroke (*P* < 0.05).

Based on these characteristics, Class I was named Mixed-Low Diabetes (Mix-LDia), Class II was named Mixed-High Diabetes (Mix-HDia), Class III was named Four-Type Hypertension (Four-HHyp), Class IV was named Three-Type Hyperuricemia, Diabetes, and Renal Dysfunction (Three-HestHDRD), Class V was named Two-Type Hyperlipidemia and Renal Dysfunction (Two-HDRD), Class VI was named One-Type Hyperlipidemia (One-HDys), and Class VII was named One-Type High Stroke (One-HApo).

### Dietary Patterns and Food Intake

Potential profile analysis was utilized in this study to classify the dietary patterns of participants, and the fitting parameters are presented in Table 3. As shown in Table 3, when comparing the Lo-Mendell-Rubin Likelihood Ratio Test (LMRT) and Bootstrap Likelihood Ratio Test (BLRT), the *P*-values for the model with three profiles were both less than 0.05. In the model with three profiles, the entropy value was 0.974, which is greater than 0.800, indicating that the classification accuracy was above 90.0% [26]. Therefore, the model with three profiles in this study was superior to others, suggesting that participants exhibited three distinct dietary patterns.

The intake of various food types among participants with different dietary patterns is detailed in Table 4. As can be seen from Table 4, there were significant differences (*P* < 0.05) in the intake of eight types of food among the different dietary patterns. Multiple comparisons revealed that, compared to participants with Pattern 2 and Pattern 3, participants with Pattern 1 had the lowest intake of cereals and cereal products, while their intake of fruits and fruit products, milk and milk products, as well as eggs and egg products, was the highest (*P* < 0.001). In contrast, participants with Pattern 2 had the lowest intake of poultry and poultry products, fruits and fruit products, as well as aquatic products, compared to those with Pattern 1 and Pattern 3 (*P* < 0.05). Additionally, compared to participants with Pattern 1 and Pattern 2, those with Pattern 3 had the highest intake of vegetables and vegetable products, as well as poultry and poultry products (*P* <0.01).

Based on the characteristics of these three dietary pattern categories, Pattern 1 was named Low Cereal High Fruit Milk and Egg Diet (LCHFM), Pattern 2 was named Low Poultry Fruit and Fishery Diet (LPFF), and Pattern 3 was named High Vegetable and Poultry Diet (HVP).

### The Impact of Dietary Patterns on CMM

The distribution of CMM among participants is detailed in Table 5. As can be seen from Table 5, participants with Class III (Four-HHyp) CMM were slightly older than those in other groups, while participants with Class VI (One-HApo) and VII (One-HApo) CMM were slightly younger than those in other groups (*P* < 0.05). Participants with Class II (Mix-HDia) and III (Four-HHyp) CMM resided in areas with higher urbanization indices. Additionally, there were associations between gender, marital status, education level, residence area, smoking status, and the categories of multiple dietary patterns, with χ^2^ values of 559.905, 95.503, 19.104, 23.942, 229.620, and 34.967, respectively (*P* < 0.01).

The impact of Dietary Patterns on CMM is detailed in Table 6. Due to the low incidence of hyperuricemia, diabetes, renal dysfunction, hypertension, and stroke in the Class VI (One-HDys, Type One Hyperlipidemia) group, the Class VI group was chosen as the reference group for comparative analysis. As shown in Table 6, compared to participants with the HVP (High Vegetable and Poultry) dietary pattern, those with the CHFM (Low Cereal High Fruit Milk and Egg) dietary pattern were more likely to have Class V (Two-HDRD, Type Two Hyperlipidemia and Renal Dysfunction) CMM *(OR=* 2.0011, 95% *CI* 1.011 – 3.960, *P* ≤ 0.05). The OR values were statistically significant in both the model controlling only for demographic characteristics and the model controlling for both demographic and lifestyle characteristics (*P* < 0.05), indicating the robustness of the model.

## Discussion

### Characteristics of Different Cardiovascular Metabolic Disease (CMM) Combination Types in Various Populations

In this study, five combined types of cardiovascular metabolic diseases (CMM, Class I to Class V) and two types of single cardiovascular or metabolic diseases (Class VI and Class VII) were identified through clustering analysis. Our findings indicate that the age of individuals with single diseases was significantly lower than those with CMM. This observation aligns with the study by Cheng et al. (2022), which reported the lowest CMM prevalence of 2.3% in the 20–19 age group and the highest of 42.9% in individuals aged ≥ 80 years [27]. Similarly, Otieno et al. (2021) found that compared to the 15–14 age group, the prevalence of CMM was higher in the 15–54 age group (PR = 3.9, 95%CI: 3.2–4.8) and the 55–69 age group (PR = 3.9, 95%CI: 3.2–4.8) [28]. Our study further reveals that participants with a higher probability of combined diabetes tend to be older than those with a lower probability. Specifically, participants with Class II (a combination of six diseases with a high probability of diabetes) were older than those with Class IV (dyslipidemia + diabetes + renal dysfunction), Class V (dyslipidemia + renal dysfunction), and Class I (a combination of six diseases with a low probability of diabetes). This suggests that dyslipidemia and renal dysfunction tend to occur earlier in middle-aged individuals, while the combination of dyslipidemia, hypertension, and diabetes emerges with advancing age. This finding is consistent with the results of a 5-year cohort study by Zhang et al. (2019), which showed that the prevalence of CMM with three-disease combinations increased nine times compared to baseline [29]. The potential reasons for this phenomenon may include the following. Dyslipidemia can lead to lipid deposition in islet cells, affecting glucose oxidation and causing β-cell apoptosis, resulting in insulin resistance. Additionally, dyslipidemia reduces glucose utilization and increases hepatic glycogenolysis, further exacerbating insulin resistance [30, 31]. Insulin resistance, in turn, triggers inflammation, oxidative stress, insulin receptor mutations, endoplasmic reticulum stress, and mitochondrial dysfunction, impairing arterial endothelial function and leading to the development of hypertension and diabetes [32, 33]. Another possible explanation is that dyslipidemia can lead to lipid deposition on blood vessel walls, causing inflammation and damaging pancreatic β-cells, interfering with glucose and lipid metabolism and leading to disorders such as insulin resistance and hyperglycemia [34, 35]. As plaques increase, the vessel diameter narrows, potentially restricting blood flow. This sequence of events explains why CMM combinations with dyslipidemia and renal dysfunction tend to occur at younger ages, while those with hypertension and diabetes occur later. Therefore, controlling dyslipidemia in middle-aged individuals plays a crucial role in managing CMM.

### The Relationship between Dietary Patterns and CMM

The current study identified three dietary patterns, Pattern I to III, through latent profile analysis (LPA). Specifically, Pattern I represents a low-cereal, high-fruit, dairy, and egg diet (LCHFM), Pattern II is a low-poultry, fruit, and fishery diet (LPFF), and Pattern III signifies a high-vegetable, poultry diet (HVP). Our findings indicate that individuals adhering to a low-grain, high-fruit, dairy, and egg diet (LCHFM) had a higher risk of developing Class V CMM (dyslipidemia + renal dysfunction) compared to those with Class VI CMM (solely dyslipidemia). This suggests that the LCHFM diet may contribute to the co-occurrence of dyslipidemia and renal dysfunction. In dietary interventions for individuals with impaired renal function, it is essential to ensure adequate intake of vegetables and dairy products. Therefore, the association between renal dysfunction and a diet low in grains and high in fruits could be attributed to several factors. Grains are a significant source of glucose, and lower glucose levels can inhibit the reabsorption of serum uric acid in the proximal tubule, leading to a reduction in serum uric acid levels and thus reducing the metabolic burden on the kidneys, ultimately protecting renal function [36–41]. Conversely, excessive fruit intake can lead to the accumulation of uric acid precursors, increasing glyconeogenesis and the pentose phosphate pathway, ultimately resulting in elevated serum uric acid levels and renal dysfunction. Dietary recommendations for individuals with dyslipidemia often involve reducing macronutrient intake, particularly carbohydrates [42]. As grains are a primary source of carbohydrates, reducing their consumption is typically advised to manage dyslipidemia. However, our findings indicate that both excessively high and low grain intake may be detrimental. Therefore, it is crucial to maintain an appropriate level of grain consumption for individuals with dyslipidemia, as both extremes may hinder disease recovery. To reduce the incidence of dyslipidemia with renal dysfunction, a balanced intake of grains, fruits, and their derivatives is essential. This study provides valuable insights into the relationship between dietary patterns and CMM, particularly in the context of dyslipidemia and renal dysfunction, and highlights the importance of tailored dietary advice for individuals with these comorbidities.

The current study has several limitations that need to be acknowledged. Firstly, the dietary data was collected through a three-day consecutive 24-hour dietary recall method, which may introduce measurement system errors compared to non-consecutive 24-hour dietary recall or food diary methods. Secondly, the analysis in this study is based on cross-sectional data, which cannot be used for causal inference. Future research should include experimental studies to draw causal conclusions and establish definitive formulations. Additionally, this study did not consider other potential factors that may influence CMM, such as genetic factors and medication use, which may have had an impact on the results. Therefore, in future studies, it is crucial to consider a wider range of potential influencing factors to arrive at more reliable conclusions.

## Conclusion

The present study identified five distinct CMM combinations: Class I, a six-disease mixture with low diabetes risk (Mix-LDia); Class II, a six-disease mixture with high diabetes risk (Mix-HDia); Class III, a four-disease combination characterized by hypertension (Four-HHyp); Class IV, a three-disease combination encompassing hyperuricemia, diabetes, and renal dysfunction (Three-HestHDRD); and Class V, a two-disease mix of hyperlipidemia and renal dysfunction (Two-HDRD). The average age of patients with these five CMM combinations decreases in the following order: Class III, Class II, Class IV, Class V, and Class I. Three dietary patterns were also discerned: Pattern I, a low-cereal, high-fruit, dairy, and egg diet (LCHFM); Pattern II, a low-poultry, fruit, and fishery diet (LPFF); and Pattern III, a high-vegetable, poultry diet (HVP). Notably, individuals adhering to the low-grain, high-fruit, dairy, and egg diet (LCHFM) pattern were found to have a higher risk of developing hyperlipidemia combined with renal dysfunction compared to those with isolated hyperlipidemia. Future studies are warranted to explore the causal relationship between dietary patterns and the progression of CMM.

## Figures and Tables

**Table 1:** Fit indices for the class 1 through 10 Models

Model	BCI^@^	ABCI^@^	ABCI ratio^@^	TDMR^@^
1	26340.205			
**2**	**20381.437**	**−5958.767**	**1**	**1.505**
3	16438.648	−3942.789	0.662	1.541
4	13897.416	−2541.232	0.426	1.026
5	11422.636	−2474.78	0.415	1.160
6	9295.428	−2127.209	0.357	1.152
**7**	**7455.201**	**−1840.227**	**0.309**	**1.883**
8	6502.069	−953.132	0.16	1.283
9	5770.153	−731.916	0.123	1.231
10	5184.983	−585.17	0.098	1.004
11	4602.436	−582.547	0.098	1.094
12	4074.075	−528.361	0.089	1.126
13	3610.409	−463.666	0.078	1.141
14	3210.486	−399.923	0.067	1.247
15	2899.954	−310.532	0.052	1.400

@ BIC, Bayesian information criterion.

ΔBCI, the change of Bayesian information criterion. ΔBCI ratio, the change ratio of Bayesian information criterion.TDMR, Target distance measurement ratio Between clusters between neighbors clusters.

**Table 2:** Disease in the classes of CMM status

Had Disease N%	*Class I Mix-LDIa*	*Class II Mix-HDia*	*Class III Four-HHyp*	*Class IV Three-HestHDRD*	*Class V Two-HDRD*	*Class VI One-HApo*	*Class VII One-HDys*	*All*	*X* ^*2*^	*P*
Hyperuricemia	781	121	0	902	0	0	0	1804	404.647	<0.001
Dyslipidemia	582	340	193	0	742	629	0	2486	3423.523	<0.001
Diabetes	2	489	0	491	0	0	0	982	4940.619	<0.001
Renal dysfunction	719	349	264	869	742	0	0	2943	3720.618	<0.001
Hypertension	121	113	435	0	0	0	0	669	3331.752	<0.001
Apoplexy	65	6	2	0	0	0	13	86	226.531	<0.001

**Table 3:** Fit indices for the profile 2 through 8 models

Profiles	LogL	AIC	BIC	aBIC	Entropy	LMRT	BLRT
2	−279373.271	558808.541	559010.344	558911.837	0.999	0.476	<0.001
3	−278082.872	556249.744	556523.154	556389.692	0.974	0.024	<0.001
4	−277106.320	554318.639	554663.657	554495.241	0.966	0.080	<0.001
5	−276295.119	552718.239	553134.864	552931.494	0.971	0.204	<0.001

^ LogL, Log likelihood.

AIC, Akaike information criterion. BIC, Bayesian information criterion. aBIC, sample size-adjusted Bayesian information criterion. BLRT, Bootstrap likelihood ratio test. LMRT, Lo-Mendell-Rubin likelihood ratio test. LogL stands for log likelihood. AIC is a metric used to compare the relative quality of statistical models, balancing model fit and complexity to select an effective model that explains data without overfitting. BIC selects models from a finite set by penalizing complexity and emphasizing model fit. BLRT compares nested models in statistical modeling. LMRT compares different parameterizations in mixture models, aiding researchers in selecting the best model for their data.

**Table 4:** Daily dietary food intakes in the latent profiles of dietary patterns

*Food intakes (mg/d)*	*All*	*Latent Dietary Patterns*			*F*	*P*
	*Mean (SD)*	*Pattern 1 LCHFM Mean (SD)*	*Pattern 2 LPFF Mean (SD)*	*Pattern 3 HVP Mean (SD)*		
Cereals and cereal products	386.852 (170.066)	297.048 (145.840)	390.395 (170.088)	409.348 (167.346)	40.742	<0.001
Tubers starches and products	30.584 (54.375)	29.804 (46.077)	31.169 (54.616)	23.833 (56.682)	2.963	0.052
Dried legumes and legume products	58.254 (75.964)	58.814 (64.550)	58.475 (76.500)	58.801 (77.158)	0.470	0.625
Vegetables and vegetable products	323.561 (172.517)	299.182 (183.426)	322.651 (171.879)	353.090 (168.753)	7.766	<0.001
Fruit and fruit products	51.786 (104.621)	133.305 (128.372)	46.093 (101.158)	62.377 (101.776)	89.970	<0.001
Meat and meat products	75.082 (70.789)	80.869 (70.677)	73.271 (70.490)	93.449 (71.953)	14.078	<0.001
Poultry and poultry products	15.442 (34.850)	21.342 (38.343)	7.120 (16.897)	115.210 (42.585)	4156.778	<0.001
Milk and milk products	13.630 (53.208)	217.787 (84.131)	2.274 (12.888)	3.834 (17.666)	10628.239	<0.001
Eggs and egg products	29.716 (37.124)	47.460 (38.866)	28.8719 (37.014)	27.082 (33.756)	31.980	<0.001
Fish shellfish and mollusc	34.696 (60.795)	52.528 (71.109)	32.165 (58.878)	53.107 (70.194)	31.251	<0.001

**Table 5:** Characteristics of the subjects in different CMM status

	CMM status								*F/X* ^*2*^	*P*
	*Mean (SD) OR N (N%)*									
	*Class I*	*Class II*	*Class III*	*Class IV*	*Class V*	*Class VI*	*Class VII*	*All*		
	*Mx-LDia*	*Mx-HDia*	*Four-HHyp*	*Three-HestHDRD*	*Two-HDRD*	*One-HDys*	*One-HApo*			
Age^	50.605 (15.029)	54.348 (14.567)	61.687 (11.439)	52.016 (14.516)	51.953 (13.919)	45.677 (15.209)	45.495 (14.699)	50.772 (15.106)	83.357	<0.001
Gender^@^ Females	592 (71.3%)	261 (53.4%)	212 (48.7%)	596 (86.6%)	496 (66.8%)	183 (29.1%)	318 (32.8%)	2658 (53.6%)	559.905	<0.001
Marital status^@^	41 (4.9%)	20 (4.1%)	1 (0.2%)	35 (4.4)	25 (3.4%)	66 (10.5%)	94 (9.7%)	282 (5.7%)	95.503	<0.001
Never married										
Education level^@^	638 (76.9%)	385 (79.1%)	343 (78.9%)	695 (80.0%)	586 (79.0%)	473 (75.2%)	705 (72.8%)	3827 (77.1%)	19.104	0.004
Below middle school										
Living areas^@^	579 (69.8%)	323 (66.1%)	265 (60.9%)	625 (71.9%)	522 (70.4%)	441 (70.1%)	699 (72.1%)	3454 (69.6)	23.942	0.001
Rural										
Urbanindex^	67.827 (19.507)	71.440 (18.648)	71.982 (19.333)	65.394 (18.766)	67.105 (18.579)	67.568 (18.449)	65.710 (18.921)	67.567 (19.007)	10.961	<0.001
PA^	118.910 (678.353)	132.500 (681.375)	127.753 (732.710)	93.924 (641.655)	71.045 (565.911)	84.837 (551.904)	75.591 (500.051)	96.717 (614.144)	1.092	0.364
Smoking status	660 (79.5%)	335 (68.5%)	286 (65.7%)	674 (77.6%)	565 (76.1%)	354 (56.3%)	531 (54.8%)	3405 (68.6%)	229.620	<0.001
Never smoke										
Dietary	44 (5.3%)	35 (7.2%)	38 (8.7%)	41 (4.7%)	41 (5.5%)	22 (35%)	38 (3.9%)	259 (5.2%)	34.967	<0.001
Pattern^@^	730 (728.5)	419 (85.7%)	365 (83.9%)	782 (90.0%)	660 (88.9%)	551 (87.6%)	849 (87.6%)	4356 (87.8%)		
***Pa*** *tt* ***e*** *r* ***n 1 LCHFM***	56 (6.7%)	35 (7.2%)	32 (7.4%)	46 (5.3%)	41 (5.5%)	56 (8.9)	82 (8.5%)	348 (7.0%)		
Pattern 2 LPFF										
***Pa*** *tt* ***e*** *r* ***n 3 HVP***										

^ The description of these variables were *Mean* (*SD*).

@ The description of these variables were *N* (*N*%).

**Table 6:** Association between dietary patterns and CMM status

## Data Availability

Data are available from https://www.cpc.unc.edu/projects/china
